# Development and Experimental Studies of an Identification Method of Kinematic Parameters for Industrial Robots without External Measuring Instruments

**DOI:** 10.3390/s22093376

**Published:** 2022-04-28

**Authors:** Anton Gubankov, Dmitry Yukhimets

**Affiliations:** 1 Laboratory of Intelligent Information Systems for Marine Robots, Institute of Marine Technology Problems, 690091 Vladivostok, Russia; 2 Robotics Laboratory, Institute of Automation and Control Processes, 690041 Vladivostok, Russia; undim@dvo.ru; 3 Department of Automation and Robotics, Far Eastern Federal University, 690091 Vladivostok, Russia; 4 Department of Informatics and Control in Technical Systems, Sevastopol State University, 299053 Sevastopol, Russia

**Keywords:** industrial robot, kinematics parameters, calibration, identification

## Abstract

This paper proposes a method for the identification of kinematic parameters of multilink series industrial robots, which does not require the use of complex and expensive equipment for high-precision external measurements of the position and orientation of an end effector in a Cartesian coordinate system. This method, by means of simple and affordable tools, enables us to achieve a significantly increased accuracy of movement of end effectors in serial robots performing various technological operations. The proposed method is experimentally verified and can be applied directly in production lines.

## 1. Introduction

In the present day, the conventional approach to industrial robots (IR) programming is manual end effector (EE) driving to required points in a working area with the help of a teach pendant. These points become base points of the EE movement trajectory considering actual location of machined parts and other additional equipment. An IR controller saves vectors of generalized coordinates (rotation angles or linear displacements of IR joints), which correspond to these base points of trajectory. Then, depending on the problem being solved, with help of various types of interpolation, the required trajectories of IR movement passing through the specified base points are formed. Accuracy of EE movement along these trajectories in automatic mode is defined by the accuracy of all actuators, which track desired values of generalized coordinates. This problem is well known, and, today, there are a lot of methods which can provide high-accuracy control of IR actuators [1,2,3,4,5,6]. 

In modern applications, IR preforms operations in uncertain working environments and generates EE trajectories offline, or by means of information from their sensors (computer vision systems, probes and other). In this case, the base points of the trajectory are set in a base coordinate system (BCS) of IR. Real positioning accuracy of EE is defined by the accuracy of the IR kinematic model, since the IR controller uses it for calculation of EE position, by means of rotation angle information or linear displacements of IR joints. This is why, if the parameters of the IR kinematic model are used in the controller differ from their real values, then the difference between the calculated EE position and its real position in BCS can be several millimeters. This is unacceptable for many technological operations [7,8,9,10,11,12,13,14]. 

There are many methods for improving IR kinematic model parameters, and many classifications of these methods are offered [15,16,17,18,19,20,21]. Fundamentally, methods of identification of IR kinematic (geometric) parameters can be divided into two groups. The first group includes methods based on using external measurement devices, and the second group doesn’t use these devices [20]. Traditionally, the most widely used methods are based on external measuring devices [15,16,17,18,19,20,21,22,23,24,25,26,27,28,29,30,31,32,33,34,35]. Previously, there were various devices for tracking EE linear movement [22], ultrasonic and infrared measurement systems [23,24], theodolite, and stereoscopic triangulation [25]. Today there are high-precision optical sensors and scanners, coordinate measuring machines (CMM), additional calibrated robots, ball bars and other precision equipment [26,27,28,29,30,31,32,33,34,35].

These methods have the following disadvantages: poor usability in real production conditions, requiring specially trained employees, requiring information about hardlyobtainable parameters, for example, a covariation matrix, and the high cost of measurement equipment. This equipment is often inaccessible for system integrators. As a result, there is the task of developing simplified procedures for the identification of IR kinematic parameters that does not require the use of expensive specialized measurement equipment. 

In [36], an identification method of geometric parameters for multilink manipulators is offered. The identification process is performed by means of EE driving to the same point. This point is a small hole on a metallic sheet. During experimental research, this point can be reached by EE with different orientations up to 40 times. This allows us to form 20 pairs of points and write 60 equations for the identification of IR parameters. As a result, the improvement parameters allow us to decrease the EE positioning error from 1 sm to 1 mm. However, experience has shown that to decrease this error to 0.1–0.3 mm, it is necessary to use about 600 pairs of points. To get such s number of points by means of a specified approach is difficult enough. Probably for this reason, this method has not been popular. 

In [37], data for the identification of IR kinematic parameters are obtained in the following manner. A special probe with known parameters is placed into holes in a calibrating plate. Requirements for the production of this probe are very high as it needs to know the position of all hole center points precisely for the forming of equations. Also, touch sensors can be used to define the contact of the probe with the hole bottom. There were 102 measurements obtained during experimental research. After performing the identification procedure and applying updated parameters in the IR kinematic model, the deviation of EE from the desired position was decreased to 0.3 mm. In the same manner, identification of kinematic parameters for UR series robots is performed. 

In [38], a method for the identification of IR kinematic parameters using a contact probe and three spheres mounted on a platform capable of changing its orientation is proposed. During the identification process, the probe is driven into 15 points of each sphere for each of five fixed orientations of platform. Data for the angles of rotation of the hinges, information about the diameter of the spheres, and the distances between them are used to adjust IR kinematic parameters. This method does not require the use of external measuring devices, but its disadvantage is the need to use additional precisely manufactured equipment.

In [39], a method is proposed for the identification of parameters for manipulators, based on minimizing the deviation of distances between points of trajectories specified in the working area, and corresponding distances were calculated using its kinematic model. At the same time, ref. [40] describes a variant of this approach using only one point of a manipulator’s working area with known coordinates. A disadvantage of this approach, one needs one or more points in the workspace, with known coordinates in the manipulator BCS. However, this is not always possible since it involves the use of external measuring devices and special procedures for setting these points. The disadvantages also include the empirical selection of correction coefficients.

In [17,41,42], similar methods are offered. In these methods, data for the identification procedure are formed by means of reaching EE to points of the same plane. In this case, data collection for identification is simpler, and can be automated. However, the result of such identification is worse than previous cases. It can be explained that the equation of the plane has four unknown parameters, and when EE reaches the point of this plane, one can only get three coordinates. To solve this problem, we must make some assumptions that may worsen identification accuracy. Moreover, we must use a precise production calibrated plane. A different perspective approach is to reach EE from points of the same surface, equation, and parameters, of which are known [43]. This surface can be produced by means of 3D printing. 

There are also a lot of confidential commercial solutions that are not published in academic journals and proceedings of conferences. These solutions are widely used in conveyers of automotive production. For example, inspection cells perform automated inspection of car bodies with high accuracy, by means of IR. To perform inspection, they have optical sensors with structured light or laser scanners. During work, IR has short and long calibration phases, which can be done in automatic mode. The general idea of such a procedure follows. Calibration artefacts are widely used. These artefacts are constructions with precisely produced spheres, pyramids and so on. Positions of, for example, sphere centers are measured by means of laser trackers. Then, after 10–20 measurements of about 30–150 points of the sphere surface by robot with different sensor orientation, and calculating its center, correction of robot parameters is proceeded by means of least mean squares method or RANSAC. Despite automatic mode, this approach is highly dependent on high-precision location of the equipment, requiring lengthy configuration and tuning, and has high cost of implementation.

Therefore, to eliminate disadvantages outlined above (the need to use precise produced calibrated equipment (plates, probes, spheres, pyramids, etc.), and external high-precision sensors for measuring position of EE in BCS) the similar procedure to the conventional method of calculating tool center point (TCP) can be applied. The data from sensors of rotation angles of actuators in all joints of IR are used in the presented procedure. This data corresponds to the rotation angles of all IR joints when EE IR reaches the same point in space with different orientations, by using a tech pedant. The method of identification of IR kinematic parameters, based on information about IR joints rotation angles will be developed. 

This paper presents an essential decrease in procedural cost for identification of IR kinematic parameters, and makes it easily accessible to a wide range of specialists (scientific staff, R&D offices, students, system integrators, production, etc.). This procedure is performed only using two probes and doesn’t need expensive external measurement devices, and additional precision equipment. These probes are used for the measurement and generation of an abundance of data for the identification of IR kinematic parameters with quality no worse than that of factory calibration. The relative simplicity of this procedure and absence of hard requirements of equipment allow us to perform identification directly on production lines. Note that the paper constitutes an extension of the conference paper [44], both theoretically and experimentally.

## 2. Problem Definition

In the present article, IR with six revolute joints and serial kinematic schemes are considered the most popular type of IR. But the proposed method can be used for any type of IR with a serial kinematic scheme. The kinematic model is described by the following equation [45]:

(1) $$T_{f}\left(\Phi ,Q\right)=\prod_{k=1}^{6}T_{k}\left(\varphi_{k},q_{k}\right),$$

where $T_{f}=\left[\begin{array}{cc} R_{f} & X_{f}\\ O & 1 \end{array}\right]\epsilon R^{4\times 4}$ is the matrix of homogeneous transformation, describing position and orientation of IR last joint (flange) in BCS *Oxyz*, located in robot base; $R_{f}\epsilon R^{3\times 3}$ is the orientation matrix of the IR flange in BCS; $X_{f}\epsilon R^{3\times 1}$ is the coordinate vector of IR flange in BCS; $O\epsilon R^{1\times 3}$ is the zero raw vector; $\Phi ={\left(\varphi_{1}^{T}\ldots \varphi_{6}^{T}\right)}^{T},\;\varphi_{k}=\left(\theta_{k},\;a_{k},d_{k},\alpha_{k}\right),\;k=\left(\bar{1,6}\right)$ is the matrix of Denavit-Hartenberg parameters; *k* is the joint number; *Q* = (*q*_1_,…, *q*_6_)^T^ is the vector of generalized coordinates (rotation angles of joints);

(2) $$T_{k}\left(\varphi_{k},q_{k}\right)=\left[\begin{array}{cccc} \cos\left(q_{k}+\theta_{k}\right) & -\sin\left(q_{k}+\theta_{k}\right)\cos\left(\alpha_{k}\right) & \;\sin\left(q_{k}+\theta_{k}\right)\sin\left(\alpha_{k}\right) & a_{k}\cos\left(q_{k}+\theta_{k}\right)\\ \sin\left(q_{k}+\theta_{k}\right) & \cos\left(q_{k}+\theta_{k}\right)\cos\left(\alpha_{k}\right) & -\cos\left(q_{k}+\theta_{k}\right)\sin\left(\alpha_{k}\right) & a_{k}\cos\left(q_{k}+\theta_{k}\right)\\ 0 & \sin\left(\alpha_{k}\right) & \cos\left(\alpha_{k}\right) & d_{k}\\ 0 & 0 & 0 & 1 \end{array}\right]\;$$

is Denavit-Hartenberg matrix [45].

The Denavit-Hartenberg representation forms a matrix of homogeneous transformations (Equation (2)) is 4 × 4 in size, and describes the position and orientation of coordinate system of the *k*-th joint respectively, the (*k* − 1)-th joint. For the definition of Equation (2), the following parameters are used: $d_{k}$ is the distance between axis $z_{k-1}$ and $z_{k}$ along the common normal; $\theta_{k}$ is the rotation angle around $z_{k-1}$ from $x_{k-1}$ to $x_{k}$; $a_{k}$ is the length of common normal; $\alpha_{k}$ is the rotation angle around common normal from $z_{k-1}$ to $z_{k}$.

The model (Equation (1)) is used for solving direct kinematic problem, i.e., for calculation of position and orientation of the IR flange in BCS on the base of values, *Q* and Φ. Moreover, for planning IR movement in BCS, the inverse kinematic problem must be solved, i.e., calculation of desired vector $Q^{*}$ corresponding desired position and orientation $T_{f}^{*}$ of IR flange:

(3) $$Q^{*}=F_{IKT}\left(T_{f}^{*},\;\Phi \right),$$

where *F_IKT_*() is function describing solution of inverse kinematic problem. Equation (3) can be implemented as an analytical expression [46] or a numerical optimization algorithm [47]. 

Controllers of IR use (Equations (1) and (3)) for calculation of current position and orientation of EE and desired rotation angles of IR actuators. Usually, matrix $\tilde{\Phi }$ of nominal geometric parameters, obtained from technical documentation, are used in these expressions. However, precise kinematic parameters Φ of specific IR can be differed from their nominal parameters $\tilde{\Phi }$ on some small values because of inaccuracies from fabrication and connection of mechanical parts:

(4) $$\Phi =\tilde{\Phi }+\Delta .$$

Using $\tilde{\Phi }$ in Equation (3) leads us to generate vector ${\tilde{Q}}^{*}$ which differ from vector $Q^{*}$ corresponding desired value $T_{f}^{*}$ on small value $\beta =Q^{*}-{\tilde{Q}}^{*}$. Presence of β leads all actuators to reach positions corresponding ${\tilde{Q}}^{*}$ and IR flange reaches position ${\tilde{T}}_{f}^{*}$ which differ from desired position $T_{f}^{*}$ on value $\varepsilon =T_{f}^{*}-{\tilde{T}}_{f}^{*}$. It means that difference of IR kinematic model parameters from real kinematic parameters of IR leads to errors in position and orientation of IR flange and EE. This is essentially important for the case when trajectory of IR movement is generated on the base of information receiving from external measurement systems (laser or optical scanners, photo cameras, and other) [7,8,9,10,11,12,13,14]. 

Improvement of IR kinematic parameters can be made by means of special measurement systems, providing high accuracy measurement of linear and angular coordinates of an end effector. But, using such a system is often impossible because of its high cost. At the same time, each IR has a high accuracy measuring system for rotation angles of its actuators that can be used for the calculation of IR kinematic parameters.

Thus, the following task is set and solved in this article. The six-degree IR, with serial kinematic scheme, is considered. Its real kinematic parameters are described by matrix **Φ**. The controller of IR uses matrix of nominal parameters $\tilde{\Phi }$ to solve direct and inverse kinematic tasks. This leads to an error *ε* of EE positioning in BCS. It is necessary to develop the method of identification of IR kinematic parameters based on series of measurements of its generalized coordinates to decrease this error.

## 3. Method of Identification of IR Kinematic Parameters

**Figure 1:**
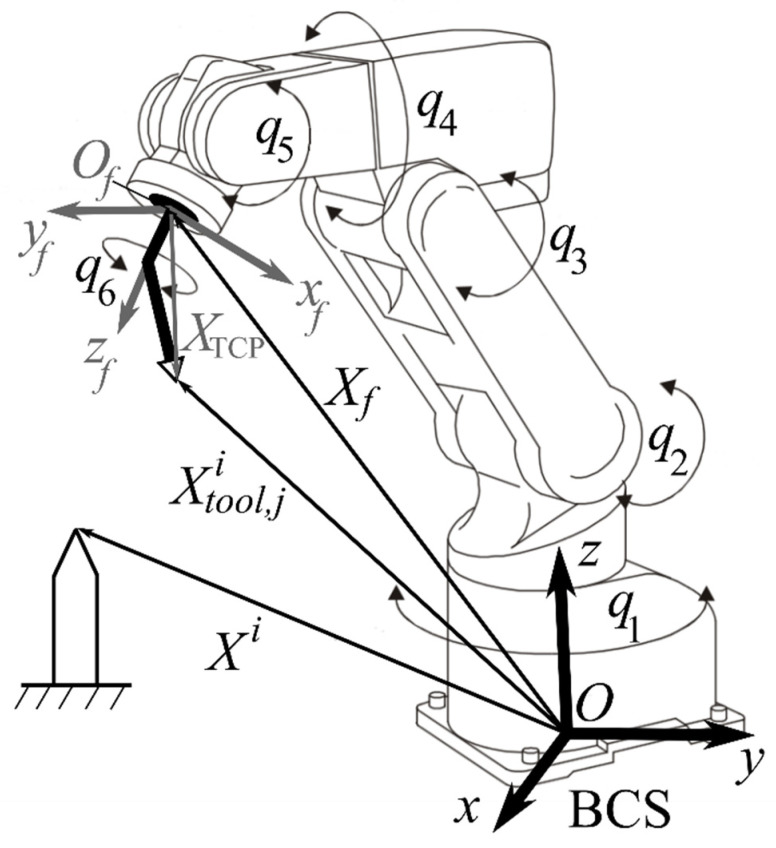
IR with serial kinematic scheme. Following notation is used: *q*_1_,…,*q*_6_ are rotation angles of IR joints; *Oxyz* is the base coordinate system; *O_f_ x_f_ y_f_ z_f_* is flange coordinate system; $X_{f}$ is the coordinate vector of the IR flange; $X_{tool,j}^{i}$ is the coordinate vector of tool center point in BSC; *X*_TCP_ is the vector of coordinates of the tool center point in *O_f_ x_f_ y_f_ z_f_*; $X^{i}$ is coordinate vector of point.

Initial data for matrix **Φ** estimation is *n* measurement series of vector Q. Each *i*-th series includes $m_{i}$ vectors $Q_{i}$, which are formed as result of EE reaching with different orientations of the same point $X^{i}$. All measurements are made manually by an operator with the help of an IR teach pendant. The $X^{i}$ position is set by a sharp probe arbitrarily located in the working space. EE movement trajectory is not important as all measurements are made in a stationary position. As a rule, for the convenience of visual inspection by the operator, a sharp probe fixed on an IR flange is used during measurement performance (see Figure 1). 

Thus, as result of measurements, is the following data array:

(5) $$\Xi =\left[\begin{array}{c} \Psi_{1}\\ \vdots \\ \Psi_{n} \end{array}\right],\;\Psi_{i}=\left(Q_{1}^{i},\ldots ,Q_{m_{i}}^{i}\right),\;i=\left(\bar{1,n}\right).$$

For each vector $Q_{j}^{i},\;i=\left(\bar{1,n}\right),\;j=\left(\bar{1,m_{i}}\right)$ we can assign vector ${\tilde{X}}_{tool,j}^{i}$ of coordinates of tool center point in BCS (see Figure 1) which calculated by means of Equation (1) and matrix $\tilde{\Phi }$:

(6) $$T_{j}^{i}\left(\tilde{\Phi },Q_{j}^{i}\right)=\left[\begin{array}{cc} {\tilde{R}}_{f,j}^{i} & {\tilde{X}}_{tool,j}^{i}\\ O & 1 \end{array}\right]=\left(\prod_{k=1}^{6}T_{k,j}^{i}\right)T_{\mathrm{TCP}},$$

where ${\tilde{R}}_{f,j}^{i}$ ∈ *R*^3×3^ is the orientation matrix of the IR flange in BCS for *j*-th measurement in *i*-th series; $T_{\mathrm{TCP}}=\left[\begin{array}{cc} E & X_{\mathrm{TCP}}\\ O & 1 \end{array}\right],$ *E* ∈ *R*^3×3^ is the unity matrix; *X*_TCP_ is the vector of coordinates of the tool center point in flange coordinate system *O_f_ x_f_ y_f_ z_f_*.

Coordinates ${\tilde{X}}_{tool,j}^{i}$, calculated by means of Equation (6), differ from coordinates of the real position of the tool center point because of the difference between used IR parameters and their real values. As EE in each measurement series reaches the same point with unknown coordinates, the real values of coordinates of TCP in the same measurement series coincided. This fact can be used for the identification of IR kinematic parameters.

Estimation of matrix $\hat{\Phi }$ of IR parameters can be made by adjusting these parameters so coordinates ${\hat{X}}_{tool,j}^{i}$ calculated by Equation (6) move closer to minimal distances. For that reason, we can use following the cost function for this estimation:

(7) $$J\left(\Xi ,\hat{\Phi }\right)=\sum_{i=1}^{n}\sum_{j=1}^{m_{i}-1}\sum_{s=j+1}^{m_{i}}{\left({\hat{X}}_{tool,j}^{i}-{\hat{X}}_{tool,s}^{i}\right)}^{2}.$$

Equation (7) doesn’t include real coordinates of points $X^{i}$ in this case for estimation of IR kinematic parameters, the high-precision measurement equipment is not needed. So, the task of identification IR kinematic parameters is formulated as follows:

(8) $$J\left(\Xi ,\;\hat{\Phi }\right)=\underset{\Phi }{\min}J\left(\Xi ,\;\Phi \right).$$

The method of numerical optimization of Levenberg-Marquardt [48] is used for the estimation of IR kinematic parameters. In this case, the initial data must be presented as follows:

(9) $$r_{p}^{i}={\hat{X}}_{tool,j}^{i}-{\hat{X}}_{tool,s}^{i},\;i=\left(\bar{1,n}\right),\;j,s=\left(\bar{1,m_{i}}\right),j<s,\;p=\left(\bar{1,l_{i}}\right),\;R_{i}=\left[\begin{array}{c} r_{1}^{i}\\ \vdots \\ r_{l_{i}}^{i} \end{array}\right],\;l_{i}=m_{i}\left(m_{i}-1\right)/2,Ρ=\left[\begin{array}{c} R_{1}\\ \vdots \\ R_{n} \end{array}\right]\in R^{3L},\;L=\sum_{i=1}^{n}l_{i}.$$

Cost function (Equation (7)) can be rewritten as follows:

(10) $$J=\frac{1}{2}\;{Ρ}^{T}Ρ.$$

The matrix Φ of IR parameters can be presented as follows:

(11) $$\vartheta =\left[\begin{array}{c} \begin{array}{c} \varphi_{1}{}^{T}\\ \vdots \\ \varphi_{6}{}^{T} \end{array}\\ X_{\mathrm{TCP}} \end{array}\right]\in R^{27}.$$

From Equation (11), we can see that 27 parameters are estimated: 24 parameters describe the IR kinematic model, and 3 parameters describe the position of TCP in the flange coordinate system *O_f_ x_f_ y_f_ z_f_*.

The initial estimate of vector ${\hat{X}}_{\mathrm{TCP}}$, we can calculate using the least squares method [49] using array Ξ. Here, we write the expression binding vector *X*_TCP_, vector $X_{f}$ position and matrix $R_{f}$ orientation of the IR flange when the probe touches its counterpart in specific points (for example, $X^{1}=X_{prob}$):

(12) $$X_{f}+R_{f}X_{\mathrm{TCP}}=X_{prob}.$$

Here, we rewrite Equation (12) as follows:

(13) $$X_{f}+\left[\begin{array}{cc} R_{f} & E \end{array}\right]\left[\begin{array}{c} X_{\mathrm{TCP}}\\ -X_{prob} \end{array}\right]=0,$$

where *E* ∈ *R*^3×3^ is unity matrix.

Equation (13) describes the model for the initial estimate of unknown vectors ${\hat{X}}_{\mathrm{TCP}}$ and ${\hat{X}}_{prob}$. To use this model, it is necessary to substitute $\tilde{\Phi }$ in Equation (1), calculating position and orientation of the IR flange for each measurement and form the following arrays:

(14) $${\tilde{X}}_{f,i}=\left[\begin{array}{c} {\tilde{X}}_{f,j}^{i}\\ \vdots \\ {\tilde{X}}_{f,m_{i}}^{i} \end{array}\right],\Omega_{i}=\left[\begin{array}{cc} {\tilde{R}}_{f,j}^{i} & E\\ \vdots & \vdots \\ {\tilde{R}}_{f,m_{i}}^{i} & E \end{array}\right],\;i=\left(\bar{1,n}\right),\;j=\left(\bar{1,m_{i}}\right).$$

The estimate of vectors ${\hat{X}}_{\mathrm{TCP}}$ and ${\hat{X}}_{prob}$ for each measurement series, we can get by means of arrays ${\tilde{X}}_{f,i}$ and $\Omega_{i}$ and least squares methods:

(15) $$\Lambda_{i}={\left(\Omega_{i}{}^{T}\Omega_{i}\right)}^{-1}\Omega_{i}{}^{T}{\tilde{X}}_{f,i},$$

where $\Lambda_{i}=\left[\begin{array}{c} {\hat{X}}_{\mathrm{TCP},i}\\ -{\hat{X}}_{prob,i} \end{array}\right]$. Here, ${\hat{X}}_{prob,i}$ is an estimate of coordinates $X^{i}$. 

The initial estimate of vector ${\hat{X}}_{\mathrm{TCP}}$ is calculated by means of expression:

(16) $${\hat{X}}_{\mathrm{TCP}}=\frac{1}{n}\sum_{i=1}^{n}{\hat{X}}_{\mathrm{TCP},i}.$$

Obtained estimate of ${\hat{X}}_{\mathrm{TCP}}$ and $\tilde{\Phi }$ are initial estimate of vector $\hat{\vartheta }$ of IR parameters Equation (11), which was used in first iteration of Levenberg-Marquardt method. Identification of $\hat{\vartheta }$ by means of this method performs by following expressions:

(17) $$\Delta \hat{\vartheta }\left(\tau \right)=-{\left[G^{T}G+\mu \left(k\right)E\right]}^{-1}G^{T}P,\;\hat{\vartheta }\left(\tau +1\right)=\{\begin{array}{c} \hat{\vartheta }\left(\tau \right)+\Delta \hat{\vartheta }\left(\tau \right),\mu \left(\tau +1\right)=\frac{\mu \left(\tau \right)}{\eta },\;\mathrm{if}\;J\left(\hat{\vartheta }\left(\tau \right)+\Delta \hat{\vartheta }\left(\tau \right)\right)<J\left(\hat{\vartheta }\left(\tau \right)\right)\\ \hat{\vartheta }\left(\tau \right),\mu \left(\tau +1\right)=\mu \left(\tau \right)\eta ,\;\mathrm{if}\;J\left(\hat{\vartheta }\left(\tau \right)+\Delta \hat{\vartheta }\left(\tau \right)\right)>J\left(\hat{\vartheta }\left(\tau \right)\right) \end{array},$$

where τ is iteration number; G=∂Ρ∂ϑ∈R3L×27; *E* ∈ *R*^27×27^ is the unity diagonal matrix; *μ*(*τ*) is a variable defined by speed of tuning; 0 < *η <* 1 is the step size adaptation parameter. The condition for finishing iteration process (Equation (17)) is $\left|J\left(\hat{\vartheta }\left(\tau \right)+\Delta \hat{\vartheta }\left(\tau \right)\right)-J\left(\hat{\vartheta }\left(\tau \right)\right)\right|<\delta$, where δ is a small positive constant. The vector P is updated according to Equation (9) on each algorithm iteration considering of improved vector $\hat{\vartheta }\left(\tau \right)$. 

As result of the work of Equation (17), the estimate of vector $\hat{\vartheta }$ is evaluated. This vector provides convergence of points ${\hat{X}}_{tool,i}^{j}$ in the *i*-th measurement series to minimal distances. Use of calculated kinematic parameters in robot controller instead its nominal parameters allow to increase accuracy of EE positioning in BCS.

## 4. Analysis of IR Parameters Identifiability and Influence of Measurement Errors on Identification Process

Further, we carry out analysis of features of identification IR parameters. Also in this analysis, we neglect indexes corresponding number of measurement series. 

Primarily, we write an expression describing dependance ${\hat{X}}_{tool}$ from kinematic parameters of IR, considering that matrix of Denavit-Hartenberg transformation for the *k*-th join has following form:

$$T_{k}=\left[\begin{array}{cc} N_{k} & L_{k}\\ O & 1 \end{array}\right],$$

where $N_{k}\in R^{3\times 3}$ is the orientation matrix of the *k*-th joint in the coordinate system of the (*k* − 1)-th joint; $L_{k}\in R^{3}$ is the position vector of the *k*-th joint in the coordinate system of the (*k* − 1)-th joint.

This dependence with considering of Equation (1) has following view:

(18) $${\hat{X}}_{tool}=L_{1}+N_{1}L_{2}+N_{1}N_{2}L_{3}+\ldots +N_{1}N_{2}N_{3}N_{4}N_{5}N_{6}L_{7}=L_{1}+\sum_{k=2}^{7}\left(\prod_{t=1}^{k-1}N_{t}\right)L_{k}.$$

Considering Equation (17), we can write:

(19) $${\hat{X}}_{tool}^{j}-{\hat{X}}_{tool}^{s}=L_{1}^{j}-L_{1}^{s}+\sum_{k=2}^{7}\left(\prod_{t=1}^{k-1}N_{t}^{j}\right)L_{k}^{j}-\sum_{k=2}^{7}\left(\prod_{t=1}^{k-1}N_{t}^{s}\right)L_{k}^{s},$$

where indexes *j* and *s* correspond to different measurements in one series. 

Considering Equation (2), the term ($L_{1}^{j}-L_{1}^{s}$) in Equation (19) has the following view:

(20) $$L_{1}^{j}-L_{1}^{s}=\left[\begin{array}{c} a_{1}\left(\cos\left(\theta_{1}+q_{1}^{j}\right)-\cos\left(\theta_{1}+q_{1}^{s}\right)\right)\\ a_{1}\left(\sin\left(\theta_{1}+q_{1}^{j}\right)-\sin\left(\theta_{1}+q_{1}^{s}\right)\right)\\ 0 \end{array}\right].$$

Parameter *d*_1_ is absent in Equation (20) and, therefore, it is absent in Equations (19) and (7). As a result, this parameter cannot be identified by the offered method. It should be noted that using an incorrect value of $d_{1}$ leads to the movement of the position of TCP along the *z* axis of BCS (see Figure 1) calculated with using this parameter, compared to its real position. In other words, the IR controller will work in coordinate systems which shifted relatively real BCS positions by small values along the *z* axis. As coordinates of points of the working area model received from the computer vision system, are recomputed to the coordinates system of the controller, then, the presence of the noted shift doesn’t influence the accuracy of reaching EE to the desired point in this coordinate system and form of EE trajectory movement. So, impossibility identification of parameter $d_{1}$ doesn’t influence accuracy in the performance of technological operations. 

Further, we consider features of parameter *θ*_1_ identification. Orientation matrix *N*_1_ presents as a multiplication of rotation matrixes on angles *θ*_1_, *q*_1_ and *α*_1_:

(21) $$N_{1}=N_{\theta 1}N_{q1}N_{\alpha 1}=N_{\theta 1}N_{1}^{\prime },$$

where *N_θ_*_1_, *N_q_*_1_ are matrixes of rotation about axis *z* of the first joint on angles *θ*_1_ and *q*_1_ respectively; *N_α_*_1_ is rotation matrix around axis *x* of the first joint on angle *α*_1_. 

Let us suppose that longitudinal axis of the first link is parallel of *z* axis of BCS, i.e., $\alpha_{1}=0$. This situation is typical for most types of IR. So, the variable $L_{1}$ considering this supposal and Equations (2) and (21) can be presented as follows:

$$L_{1}=N_{\theta 1}N_{q1}^{\prime }A_{1},$$

where *A*_1_ = [*a*_1_, *a*_1_, *d*_1_]*^T^* (see Equation (2)), and $X_{tool}$, therefore, in form:

(22) $$X_{tool}=N_{\theta 1}N_{1}^{\prime }A_{1}+N_{\theta 1}N_{1}^{\prime }\sum_{k=2}^{7}\left(\prod_{t=2}^{k-1}N_{t}\right)L_{k}.$$

Considering Equation (22), Equation (19) can be represented as follows:

(23) $${\hat{X}}_{tool}^{j}-{\hat{X}}_{tool}^{s}=N_{\theta 1}\left({N^{\prime }}_{1}^{j}\left(A_{1}+\sum_{k=2}^{7}\left(\prod_{t=2}^{k-1}N_{t}^{j}\right)L_{k}^{j}\right)-{N^{\prime }}_{1}^{s}\left(A_{1}+\sum_{k=2}^{7}\left(\prod_{t=2}^{k-1}N_{t}^{s}\right)L_{k}^{s}\right)\right).$$

From Equation (23), we see that turning on *θ*_1_ does not affect value $\left|\left|{\hat{X}}_{tool}^{j}-{\hat{X}}_{tool}^{s}\right|\right|$ as multiplication by the matrix *N_θ_*_1_ provides only rotation of coordinates of all points of ${\hat{X}}_{tool}$ on angle *θ*_1_ along the *z*-axis. This rotation will not affect value of the Equation (7), which makes it impractical to tune specified parameter by proposed method.

Next, consider situation when parameter *α_k_* = 0, that is, two joints *k* and *k* + 1 have axes of rotation located parallel to each other. In this case, orientation matrices of coordinate systems of these joints in BCS will be described as follows:

(24) $$U_{k}=\prod_{t=1}^{k}N_{t}=\left[\begin{array}{ccc} u_{11} & u_{12} & u_{13}\\ u_{21} & u_{22} & u_{23}\\ u_{31} & u_{32} & u_{33} \end{array}\right],\;U_{k+1}=U_{k}N_{k+1}=\left[\begin{array}{ccc} u_{11} & u_{12} & u_{13}\\ u_{21} & u_{22} & u_{23}\\ u_{31} & u_{32} & u_{33} \end{array}\right]\left[\begin{array}{ccc} c_{k} & -s_{k} & 0\\ s_{k} & c_{k} & 0\\ 0 & 0 & 1 \end{array}\right]=\left[\begin{array}{ccc} c_{k}u_{11}+s_{k}u_{12} & c_{k}u_{12}-s_{k}u_{11} & u_{13}\\ c_{k}u_{21}+s_{k}u_{22} & c_{k}u_{22}-s_{k}u_{21} & u_{23}\\ c_{k}u_{31}+s_{k}u_{32} & c_{k}u_{32}-s_{k}u_{31} & u_{33} \end{array}\right],$$

where $c_{k}=\cos\left(\theta_{k}+q_{k}\right)$, $s_{k}=\sin\left(\theta_{k}+q_{k}\right)$.

The coordinate vectors of position of *k* and *k* + 1 joints are determined by the expressions:

(25) $$L_{k}=U_{k}\left[\begin{array}{c} a_{k}c_{k}\\ a_{k}s_{k}\\ d_{k} \end{array}\right],\;L_{k+1}=U_{k+1}\left[\begin{array}{c} a_{k+1}c_{k+1}\\ a_{k+1}s_{k+1}\\ d_{k+1} \end{array}\right].$$

From Equation (17), it can be seen that partial derivatives of the vector *P* against *d_k_* and *d_k_*_+1_, taking into account Equations (19) and (24), will have the form:

(26) $$\frac{\partial \left({\hat{X}}_{tool}^{j}-{\hat{X}}_{tool}^{s}\right)}{\partial d_{k}}=U_{k}^{j}\frac{\partial L_{k}^{j}}{\partial d_{k}}-U_{k}^{s}\frac{\partial L_{k}^{s}}{\partial d_{k}}=\left(U_{k}^{j}-U_{k}^{s}\right)\left[\begin{array}{c} 0\\ 0\\ 1 \end{array}\right]=\left[\begin{array}{c} u_{13}^{j}-u_{13}^{s}\\ u_{23}^{j}-u_{23}^{s}\\ u_{33}^{j}-u_{33}^{s} \end{array}\right],\;\frac{\partial \left({\hat{X}}_{tool}^{j}-{\hat{X}}_{tool}^{s}\right)}{\partial d_{k+1}}=U_{k+1}^{j}\frac{\partial L_{k+1}^{j}}{\partial d_{k+1}}-U_{k+1}^{s}\frac{\partial L_{k+1}^{s}}{\partial d_{k+1}}=\left(U_{k+1}^{j}-U_{k+1}^{s}\right)\left[\begin{array}{c} 0\\ 0\\ 1 \end{array}\right]=\left[\begin{array}{c} u_{13}^{j}-u_{13}^{s}\\ u_{23}^{j}-u_{23}^{s}\\ u_{33}^{j}-u_{33}^{s} \end{array}\right].$$

As it can be seen from Equation (26), the partial derivatives for parameters *d_k_* and *d_k_*_+1_ for case *α_k_* = 0 have the same values. This means that, in this case, it is not possible to separate these parameters, since they will change in the same manner regardless of their actual values. This situation occurs when calculating parameters *d*_2_ and *d*_3_ for PUMA type IR, as well as for a flange with a tool attached to it.

Next, we will consider the effect on the identification process of tool driving errors to the same point in space with different orientations. These errors appear due to physiological characteristics of the human operator, who cannot visually determine approach TCP to a given point, with an error of less than 0.1 mm. In this case, additional errors will be present in the set of measurements, which do not depend on accuracy of kinematic model adjustment. That is, Equation (7) can be rewritten as:

(27) $$J\left(\Xi ,\hat{\Phi }\right)=\sum_{i=1}^{n}\sum_{j=1}^{m_{i}-1}\sum_{s=j+1}^{m_{i}}{\left({\hat{X}}_{tool,j}^{i}+\zeta_{j}^{i}-\left({\hat{X}}_{tool,s}^{i}+\zeta_{s}^{i}\right)\right)}^{2},$$

where the indices *j* and *s* correspond to different measurements in the *i*-th series; $\zeta_{j}^{i}$ is the tool driving errors to a given point during the *j*-th measurement in the *i*-th series; ${\hat{X}}_{tool,j}^{i}$ are the coordinates of the tool calculated by Equation (6) using vector ${\hat{Q}}_{j}^{i}$, corresponding to the angles of IR joints rotation when it is accurately driven to a given point in the *j*-th measurement in the *i*-th series. That is, vector $Q_{j}^{i}$ can be represented as $Q_{j}^{i}={\hat{Q}}_{j}^{i}+E_{j}^{i}$, and the occurrence of errors $\zeta_{j}^{i}$ are due to the presence of the value $E_{j}^{i}$. Obviously, in the presence of $\zeta_{j}^{i}$, Equation (27) will not be equal to 0, even with the fine-tuning of the IR parameters, when $\left|\left|{\hat{X}}_{tool,j}^{i}-{\hat{X}}_{tool,s}^{i}\right|\right|=0$. Next, we consider how tool driving errors will affect the process of identification of IR parameters.

Taking *E* into account, leading to tool driving errors to a given point, and Equation (2), it is possible to write:

(28) $$L_{k}=\left[\begin{array}{c} a_{k}\cos\left(q_{k}+\theta_{k}+e_{k}\right)\\ a_{k}\cos\left(q_{k}+\theta_{k}+e_{k}\right)\\ d_{k} \end{array}\right],$$

where $e_{k}$ is corresponding entry of vector E.

It can be seen from Equation (19) that it is possible to reduce the difference between measurements at the presence of the tool driving errors only if the values $L_{k}$ will be reduced. That is, if this will be possible by means of reducing modulus of IR linear parameters (see Equation (28)). Thus, after the initial reduction of Equation (27) due to turning of the majority (except described above, see Equations (20), (23) and (26)) IR parameters, further reduction of its value, which includes $\zeta_{j}^{i}$, is possible by reducing the linear parameters $a_{k}$ and $d_{k}$. As a result, if the specified process is not limited, then parameters obtained during the identification parameters will no longer correspond to real ones.

To reduce the influence of $\zeta_{j}^{i}$ on the process of identification in vector *P* (see Equation (9)), it is necessary to include only those measurements that satisfy the condition $||r_{p}^{i}||\geq \Upsilon$, where Υ is an estimate of the tool driven accuracy to a given point. The value of Υ is chosen empirically, and its value, in most cases, can be assumed to be 0.1 mm.

## 5. Simulation Results

Numerical simulation was carried out to verify the proposed method for identification of IR kinematic parameters. In the simulation, the Mitsubishi RV-2FB robot was considered, with a PUMA kinematic scheme (see Figure 1).

The matrix of nominal parameters of Dennavit-Hartenberg and the matrix of deviations of these parameters from the nominal ones had the form:

$$\tilde{\Phi }=\left[\begin{array}{cccc} \theta ,\;{}^{\circ} & a,\;\mathrm{mm} & d,\;\mathrm{mm} & \alpha ,\;{}^{\circ}\\ 0 & 0 & 295 & -90\\ -90 & 230 & 0 & 0\\ \begin{array}{c} -90\\ \begin{array}{c} 0\\ 0\\ 180 \end{array} \end{array} & \begin{array}{c} 50\\ \begin{array}{c} 0\\ 0\\ 0 \end{array} \end{array} & \begin{array}{c} 0\\ \begin{array}{c} 270\\ 0\\ 70 \end{array} \end{array} & \begin{array}{c} -90\\ \begin{array}{c} 90\\ -90\\ 0 \end{array} \end{array} \end{array}\right],\;\;\Delta =\left[\begin{array}{cccc} \theta ,\;{}^{\circ} & a,\;\mathrm{mm} & d,\;\mathrm{mm} & \alpha ,\;{}^{\circ}\\ 0 & 0.4 & 0 & 0.115\\ 0.115 & -0.4 & 0.4 & -0.17\\ \begin{array}{c} 0\\ \begin{array}{c} -0.17\\ 0.115\\ 0 \end{array} \end{array} & \begin{array}{c} 0.3\\ \begin{array}{c} 0.2\\ 0.3\\ 0.2 \end{array} \end{array} & \begin{array}{c} 0\\ \begin{array}{c} 0.3\\ 0.2\\ 0.3 \end{array} \end{array} & \begin{array}{c} 0.23\\ \begin{array}{c} 0.17\\ -0.115\\ 0 \end{array} \end{array} \end{array}\right].\;$$

**Figure 2:**
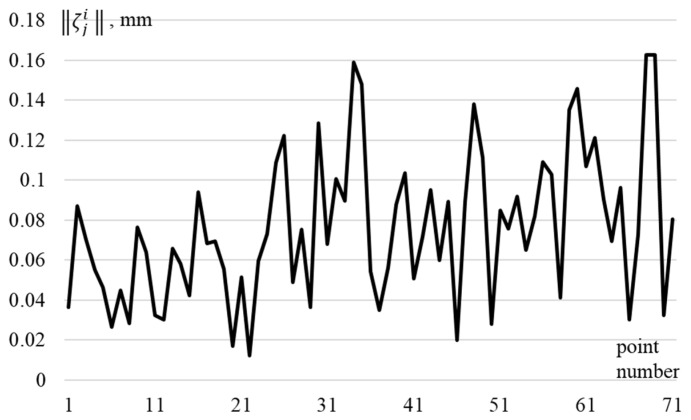
Values of tool driving errors $||\zeta_{j}^{i}||$ during the *j*-th measurement in the *i*-th series.

To test the proposed method, two arrays of data, $\Xi_{0}$ and $\Xi_{\zeta }$, containing four series of measurements were generated. They correspond to the approach of the EE *X*_TCP_ = [10, 20, 109]^T^ with different orientation to points $X^{1}$ = [7, −317, 106]^T^, $X^{2}$ = [−366, 10, 106]^T^, $X^{3}$ = [20, 300, 106]^T^, and $X^{4}$ = [200, 200, 106]^T^. The first array $\Xi_{0}$, corresponded to the case when there were no errors $\zeta_{j}^{i}$, and the second array corresponded to the case when these errors were formed randomly, while their amplitude did not exceed 0.16 mm (see Figure 2).

Next, using the matrix of nominal parameters $\tilde{\Phi }$ and arrays $\Xi_{0}$, $\Xi_{\zeta }$ according to Equations (14)–(16), the vector ${\hat{X}}_{\mathrm{TCP}}$ was calculated. For the first case, when there are no errors: ${\hat{X}}_{\mathrm{TCP}}^{0}$ = [10.25, 19.77, 109.25]^T^, for the second case ${\hat{X}}_{\mathrm{TCP}}^{\zeta }$ = [10.26, 19.78, 109.25]^T^.

The calculated data was used to identify kinematic parameters by the proposed method. An initial value of *J* for the array $\Xi_{0}$ was 728, and the final value after nine iterations of tuning was 2.5 × 10^−4^. For the array $\Xi_{\xi }$, the initial value was 732, and the final value after four iterations of tuning was 8.16.

As a result of tuning, the following IR parameter values were obtained:

$${\hat{\Phi }}_{0}=\left[\begin{array}{cccc} \theta ,\;{}^{\circ} & a,\;\mathrm{mm} & d,\;\mathrm{mm} & \alpha ,\;{}^{\circ}\\ 0 & 0.399 & 295 & -89.89\\ -89.88 & 239.61 & 0.2 & -0.17\\ -90 & 50.3 & 0.2 & -89.77\\ 0.172 & 0.2 & 270.31 & 90.17\\ 0.115 & 0.3 & 0.2 & -90.115\\ 180.22 & 0.005 & 69.95 & -0.078 \end{array}\right],\;\;{\hat{\Phi }}_{\zeta }=\left[\begin{array}{cccc} \theta ,\;{}^{\circ} & a,\;\mathrm{mm} & d,\;\mathrm{mm} & \alpha ,\;{}^{\circ}\\ 0 & 0.47 & 295 & -89.89\\ -89.87 & 239.6 & 0.17 & -0.166\\ -90.00 & 50.21 & 0.17 & -89.77\\ -0.17 & 0.2 & 270.28 & 90.17\\ 0.124 & 0.33 & 0.16 & -90.03\\ 180.2 & 0 & 69.97 & -0.05 \end{array}\right]\;{\hat{X}}_{\mathrm{TCP}}^{0}={\left[10.28,\;19.81,\;109.38\right]}^{T},\;{\hat{X}}_{\mathrm{TCP}}^{\zeta }={\left[10.28,\;19.82,\;109.38\right]}^{T}.$$

As can be seen from the presented results, without tool driving errors at a given point, the proposed method provides a high accuracy for parameter identification (the error in identifying linear parameters does not exceed 0.01 mm, and angular parameters 0.001°). At the same time, if there are specified errors in the measurement array, the accuracy of parameter identification decreases (the error in identification of linear parameters does not exceed 0.07 mm, and angular parameters 0.02°).

In addition, from the presented results it can be seen that the parameters *d*_2_ and *d*_3_ have the same value, and their sum corresponds to the real value of the parameter *d*_2_. Also, the parameters of TCP and the sixth link of the robot are identified together and are not separated. At the same time, the obtained parameters ${\hat{X}}_{\mathrm{TCP}}$ and ${\hat{\varphi }}_{6}$ differ from the reference ones, but they allow us to accurately determine the position of the tool. The *z* coordinate of the tool, relative to the sixth link, is determined by the sum of *d*_6_ and the *z*_TCP_ coordinate in the *X*_TCP_ vector, and the *x* and *y* coordinates by the expressions: $x=a_{6}c_{6}+x_{\mathrm{TCP}},\;y=a_{6}s_{6}+y_{\mathrm{TCP}}$. In the real case, when working tools will be installed instead of a probe after identification, it is recommended to carry out a standard procedure for determining TCP, considering the identified parameters. 

**Figure 3:**
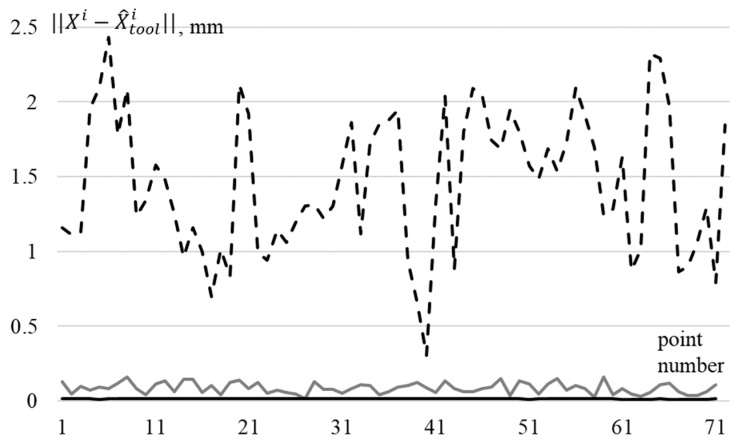
Deviations of TCP position ${\hat{X}}_{tool}^{i}$, calculated according to the kinematic model of the IR using various sets of parameters, from the real position $X^{i}$.

Figure 3 shows the deviation of the TCP coordinates calculated according to the model, Equation (6), using the identified parameters $\hat{\Phi }$ from their true values (solid black line is the deviation when using parameters ${\hat{\Phi }}_{0}$, gray is when using parameters ${\hat{\Phi }}_{\zeta }$, and dotted when using nominal parameters $\tilde{\Phi }$).

It can be seen from Figure 3 that identified parameters allow for the calculating position of EE with an accuracy of 0.016 mm, if the measurements were carried out without errors, and with an accuracy of 0.16 mm, if the measurements were carried out with errors, as shown in Figure 2. This accuracy is sufficient to perform most of basic technological operations. Figure 3 shows that when using nominal parameters of IR, accuracy of determining the position of the tool drops sharply, and the error can reach 2.4 mm, which is unacceptable when performing critical technological operations.

Thus, the results of the simulation confirmed operability and effectiveness of the proposed method for identification of IR kinematic parameters. It should be noted that use of criterion, Equation (7), makes it possible to obtain 628 differences for the parameter identification from four series of measurements at four stationary points $m_{i}=\left(21,\;21,\;18,\;11\right)$. This explains its high accuracy.

## 6. Experimental Research

### 6.1. Experiment Description

**Figure 4:**
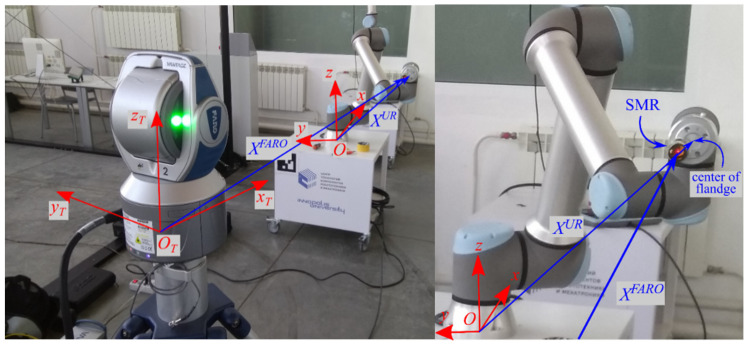
Experimental setup.

The purpose of the experiment is to verify the method described above. During the verification process, it was necessary to evaluate the accuracy of the calculating position of IR flange center using kinematic parameters obtained during the identification process. For this purpose, a laser tracker was used. It allows us to determine the coordinates of the center of spherically mounted rertroreflectors (SMR) with high accuracy in the associated coordinate system $O_{T}x_{T}y_{T}z_{T}$. The laboratory setup is shown in Figure 4.

When using a tracker to verify this method, there are two main problems. The first is the mismatch of the coordinate systems *Oxyz* and $O_{T}x_{T}y_{T}z_{T}$, which does not allow direct comparison of the coordinates measured by tracker with calculated coordinates of the IR flange position. The second is the mismatch of the center of the SMR, that is, the point whose position tracker measures, with the center of IR flange (see Figure 4), whose coordinates are calculated according to Equation (1) using the identified parameters.

These problems were solved as follows.

To perform the comparison of coordinates *X^FARO^* measured by tracker with coordinates ${\hat{X}}^{UR}$ of flange calculated by model, Equation (1), using parameters identified by the proposed method, it is necessary to make a series of measurements, after which, using the ICP (Iterative Closest Points) algorithm, set of coordinates ${\hat{X}}^{UR}$ (cloud of points) should be aligned with cloud *X^FARO^*. This will provide an alignment of IR coordinate system with the tracker. Accuracy of the IR kinematic model containing identified parameters will be estimated by the standard deviation between the points of these clouds after their alignment. The smaller this value, the more precisely the kinematic parameters of the IR are estimated. 

In order to exclude the influence of mismatch of SMR and flange central point, all movements of the IR during the measurement were carried out with the same flange orientation. In this case, the measured coordinates of SMR will always be shifted by the same value, relative to the center of flange. 

Thus, experimental studies of the method of identification for IR kinematic model parameters consisted of the following stages.

**Figure 5:**
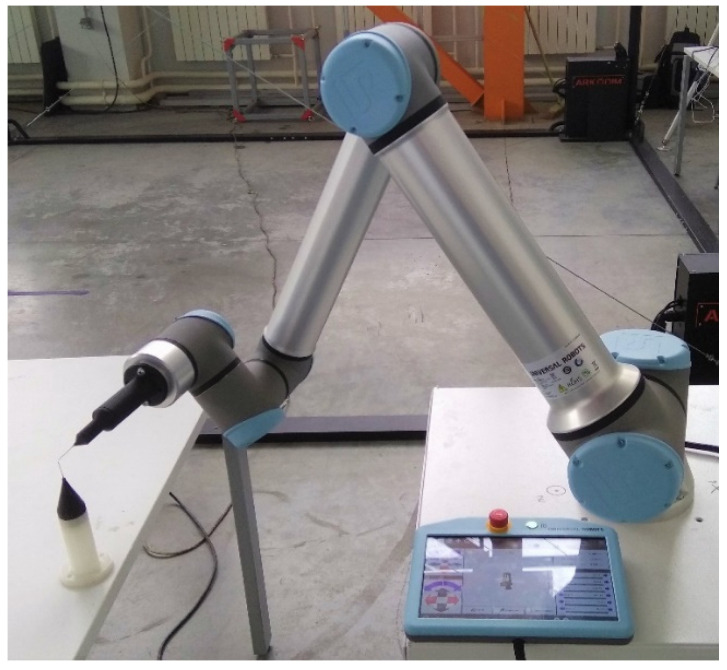
Process of measurements.

At the first stage, data was collected. Twenty-one measurements were performed manually at three fixed points in space *X*^1^–*X*^3^ (see Figure 5) and array **Ξ**. At the second stage, the kinematic parameters of the considered robot were identified based on obtained data. That is, the matrix $\hat{\Phi }$ was evaluated.

At the third stage, positions of SMR *X^FARO^*, mounted on IR flange, were measured in $O_{T}x_{T}y_{T}z_{T}$ by means of a laser tracker (see Figure 4). Then, the flange was moved, and measurements were performed again. Since SMR was not fixed in the geometric center of flange, its orientation remained unchanged, that is, only linear movements of flange were performed.

Simultaneously with recording positions of SMR, the following data was also stored: positions of flange center *X^UR^* in BCS *Oxyz*, position calculated by IR controller, and corresponding values of generalized coordinates *Q_UR_*. Based on the *Q_UR_*, according to Equation (1), coordinates ${\tilde{X}}^{UR}$ of flange center in BCS *Oxyz* were calculated with the help of parameters from the technical documentation $\tilde{\Phi }$, as well as coordinates ${\hat{X}}^{UR}$ evaluated using parameters $\hat{\Phi }$, identified by the proposed method. Thus, during the measurement process, four point clouds *X^FARO^*, *X^UR^*, ${\tilde{X}}^{UR}$, ${\hat{X}}^{UR}$ were obtained.

At the fourth stage, for the convenience of further analysis and simplification of the procedure of coordinate system alignment using the ICP algorithm, all geometric centers of point clouds were transferred to the beginning of the coordinate system. To do this, the coordinates of all points in each cloud were recalculated using formula

$$x_{p}=x_{p}-\frac{1}{P_{x}}\sum_{p=1}^{P_{x}}x_{p},\;p=\left(\bar{1,P_{x}}\right),$$

where *x_p_* is the coordinates of a specific point in the point cloud; *P_x_* is the number of points in this cloud.

Finally, at the fifth stage, point clouds were aligned using the well-known ICP algorithm. Moreover, clouds *X^UR^*, ${\tilde{X}}^{UR}$ and ${\hat{X}}^{UR}$ were moved to *X^FARO^*. Then, by the deviation of points of three clouds *X^UR^*, ${\tilde{X}}^{UR}$ and ${\hat{X}}^{UR}$ from points *X^FARO^* closest to them and by the total standard deviation, one can indirectly conclude quality of identified parameters. The smaller standard deviation of points after the aligning procedure, the less these points deviate from accurately measured *X^FARO^* and the more accurate the kinematic parameters of model, Equation (1), were identified.

### 6.2. Results of Experimental Research

During experimental studies, six degrees-of-freedom IR UR10e with serial kinematic scheme, other than PUMA scheme, and FARO Vantage laser tracker were used. Measurement accuracy of the tracker is up to 0.015 mm (see Figure 4).

In the first stage, data was collected to identify parameters of IR. The probe was driven manually using a teach pendant, and accuracy of this probe driving to given point was inspected visually (see Figure 5). Twenty-one (*m*_1_ = *m*_2_ = *m*_3_ = 21) measurements were performed at three fixed points in space (*i* = 3). At the same time, vectors $Q_{j}^{i}$ were stored, and thereby, data set **Ξ** was formed.

At the second stage, identification of IR parameters was performed. The initial value of *J*, calculated using Equation (7), was 1120, and, after three iterations, it was 28.7. The matrices of identified and nominal parameters had following form:

$$\hat{\Phi }=\left[\begin{array}{cccc} \theta ,{}^{\circ} & a,\mathrm{mm} & d,\mathrm{mm} & \alpha ,{}^{\circ}\\ 0 & 0.0015 & 180.7 & 90.0193\\ 0.0047 & -612.7056 & -0.0002 & -0.0682\\ 0.0015 & -571.3064 & -0.0002 & -0.2073\\ 0.0017 & -0.0756 & 174.1 & 89.9643\\ 0.0467 & 0.1869 & 120.2671 & -90.0688\\ -0.0253 & 0.0356 & 115.8641 & -0.0102 \end{array}\right],\;\tilde{\Phi }=\left[\begin{array}{cccc} \theta ,{}^{\circ} & a,\mathrm{mm} & d,\mathrm{mm} & \alpha ,{}^{\circ}\\ 0 & 0 & 180.7 & 90\\ 0 & -612.7 & 0 & 0\\ 0 & -571.5 & 0 & 0\\ 0 & 0 & 174.1 & 90\\ 0 & 0 & 119.8 & -90\\ 0 & 0 & 116.5 & 0 \end{array}\right].$$

**Figure 6:**
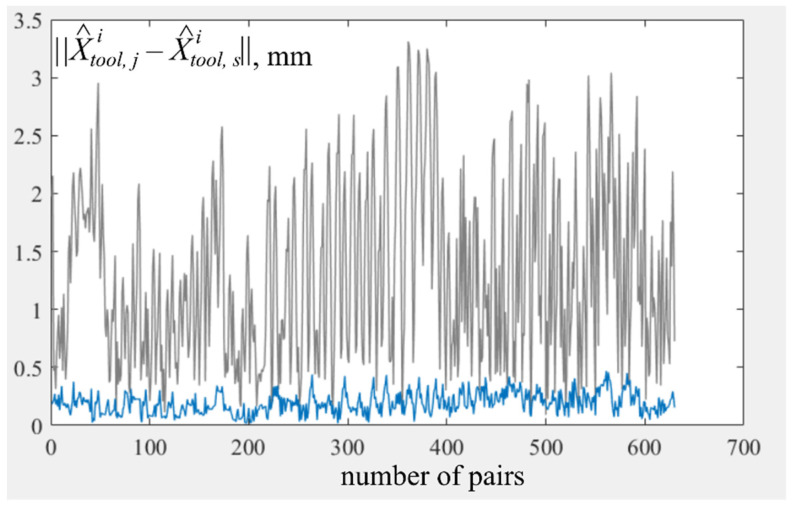
Distances between all pairs of points, indexes *j* and *s* are corresponded to different measurements in series.

Figure 6 shows distances between all pairs of points (there were 630 of them), whose coordinates were calculated based on **Ξ** using Equation (1), and matrices $\tilde{\Phi }$ (gray graph) and $\hat{\Phi }$ (blue graph), respectively. From this Figure, it can be seen that use of $\hat{\Phi }$, estimated as proposed in this paper, makes it possible to reduce the distances in each of pairs of points in the *X*^1^–*X*^3^ measurement series by more than ten times.

**Figure 7:**
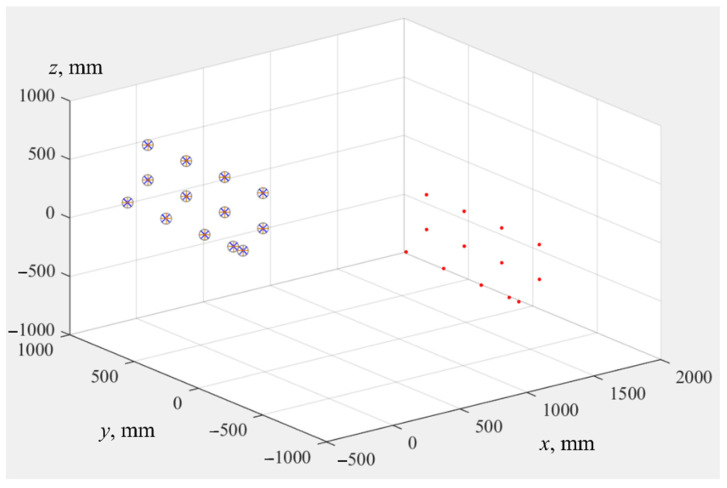
Point clouds: *X^FARO^* are the positions of SMR measured in $O_{T}x_{T}y_{T}z_{T}$ by means of laser tracker (red dots), *X^UR^* are positions of flange center in *Oxyz*, calculated by IR controller (orange plus signs), ${\tilde{X}}^{UR}$ are coordinates of flange center in *Oxyz*, calculated with help of parameters from the technical documentation $\tilde{\Phi }$ (gray circles), ${\hat{X}}^{UR}$ are coordinates of flange center in *Oxyz*, evaluated using parameters $\hat{\Phi }$ (blue crosses).

At the third stage, coordinates of all point clouds (*X^FARO^*, *X^UR^*, ${\tilde{X}}^{UR}$, ${\hat{X}}^{UR}$) were obtained. Their values are given in Table 1. All of 13 points were measured by the tracker, shown as red dots in Figure 7. For clarity, in the same coordinate system, calculated points are shown: ${\hat{X}}^{UR}$ are indicated by blue crosses, ${\tilde{X}}^{UR}$ are gray circles, *X^UR^* are orange plus signs.

**Figure 8:**
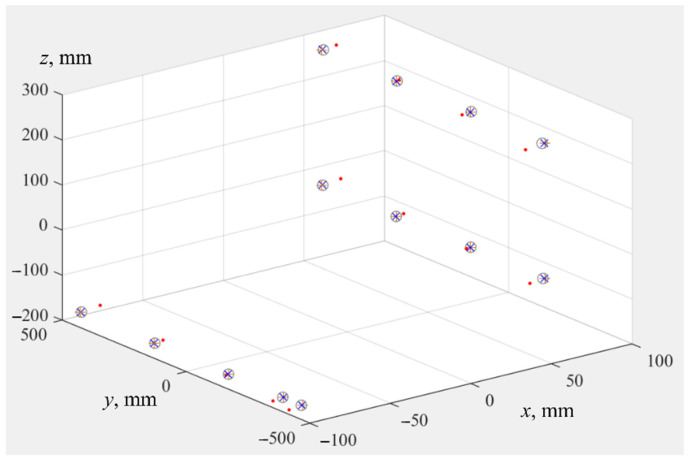
Initial position of point clouds before registration. *X^FARO^* are red dots, ${\hat{X}}^{UR}$ are blue crosses, ${\tilde{X}}^{UR}$ are gray circles, *X^UR^* are orange plus signs.

In the fourth stage, geometric centers of all point clouds were calculated, and clouds were moved to the beginning of the coordinate system. The result of this transfer is shown in Figure 8. This figure shows a mismatch of axes of coordinate systems of IR *Oxyz* and tracker $O_{T}x_{T}y_{T}z_{T}$, as well as differences caused by use of different sets of IR parameters.

**Figure 9:**
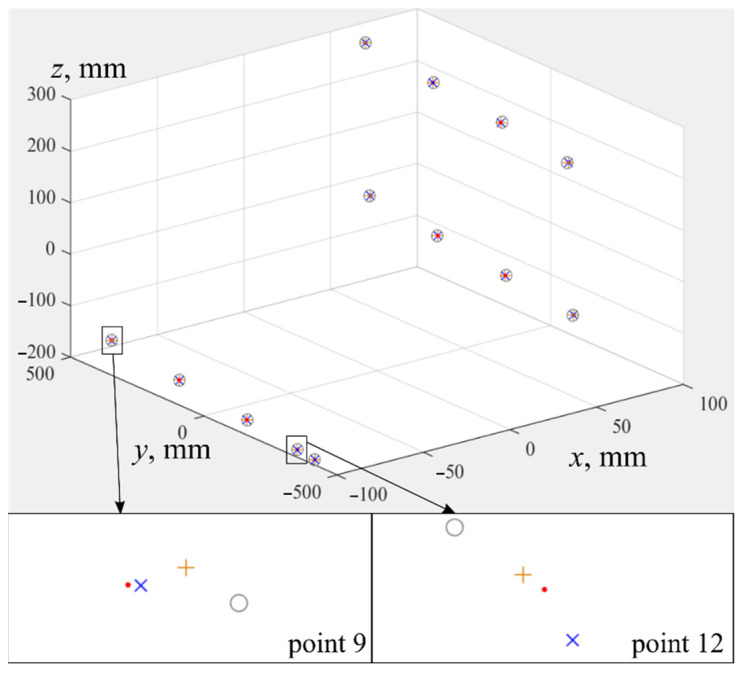
Position of point clouds after registration. *X^FARO^* are red dots, ${\hat{X}}^{UR}$ are blue crosses, ${\tilde{X}}^{UR}$ are gray circles, *X^UR^* are orange plus signs.

In the last stage, point clouds were aligned by their initial position shown in Figure 8. Its result is shown in Figure 9. This alignment was performed in CloudCompare program, which uses an open PLC library. As a result, standard deviation took the following values: when aligning *X^FARO^* and ${\hat{X}}^{UR}$, it was 0.2262 mm, *X^FARO^* and *X^UR^* was 0.2562 mm, and *X^FARO^* and ${\tilde{X}}^{UR}$ was 0.6715 mm.

**Figure 10:**
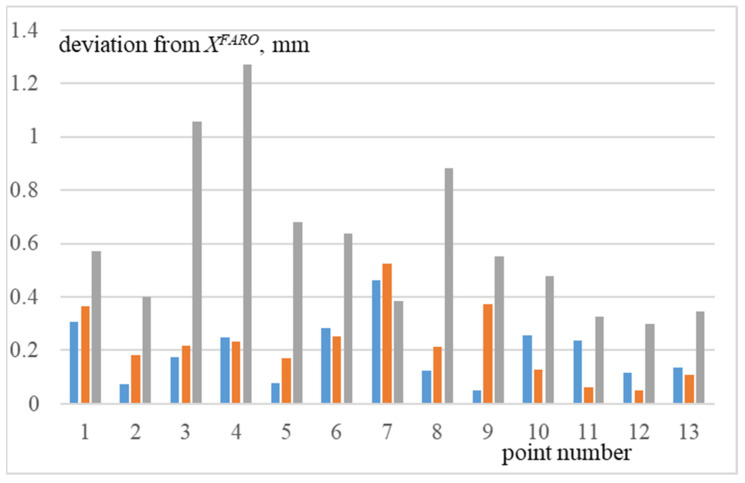
Deviation of points from *X^FARO^*. Blue indicates deviation from *X^FARO^* points of cloud ${\hat{X}}^{UR}$, orange is *X^UR^*, gray is ${\tilde{X}}^{UR}$.

Deviation of each of the cloud points, *X^UR^*, and ${\tilde{X}}^{UR}$ from corresponding points *X^FARO^* is shown in Figure 10. Blue in Figure 10 indicates deviation from *X^FARO^* points of cloud ${\hat{X}}^{UR}$, orange is *X^UR^*, gray is ${\tilde{X}}^{UR}$.

Thus, parameters identified by the proposed method best approximate the coordinates of points calculated with their help to ones obtained by means of the high-precision laser tracker. That is, the proposed method provided result no worse, and even a little better, than calibration of UR10e robot performed in factory conditions with the help of special precision tools (calibration plate or another calibrated robot). As a result, performed experimental studies have fully confirmed the operability and effectiveness of this method for the identification of IR parameters without use of external measuring devices.

## 7. Conclusions

The paper proposes a method for the identification of kinematic parameters of IR with a series kinematic scheme, which does not require use of external high-precision measuring systems. The proposed method consists of two stages. At the first stage, this is done manually by means of a teach pendant, driven at different orientations to the same fixed point in space and data on the rotation angles of actuators is recorded. At the second stage, using the Levenberg-Marquardt method, the IR model kinematic parameters are tuned in such way as to reduce distances between pairs of tool positions calculated based on mathematical model of this IR. As a result of proposed procedure, it is possible to refine kinematic parameters and, as the research results have shown, significantly increase the accuracy of IR movement in BCS. Experimental studies of the proposed method were carried out and they confirmed its operability and effectiveness. 

The proposed method has the following restrictions: it can be used for IR with a serial kinematic scheme, it needs a teach pendant, and an operator, automatization is hardly implemented. However, it can also be used to solve the problem of identifying the elastostatic parameters of IR, which is still relevant. In [50], the results of its use are described, simulation is performed. Experimental research is currently being carried out.

## Figures and Tables

**Table 1:** Coordinates for all points in clouds.

***X^FARO^***			***X^UR^***		
***x*, mm**	***y*, mm**	***z*, mm**	***x*, mm**	***y*, mm**	***z*, mm**
1541.2800	349.3100	−78.6300	−300.0089	599.9986	749.9492
1534.1100	49.1100	−80.9600	−299.9971	300.0101	749.9022
1527.0700	−250.9700	−83.5100	−299.9978	0.0120	749.9403
1520.3200	−550.8800	−86.2700	−299.9792	−299.9835	749.9475
1523.4300	−548.3200	−386.0500	−300.0052	−299.9906	450.0485
1530.0900	−248.4840	−383.4900	−299.9870	0.0142	450.0200
1537.1500	51.05500	−380.8400	−299.9926	299.9972	450.0468
1544.3600	351.6540	−378.4650	−299.9857	599.9974	450.0407
1396.0000	356.5200	−530.1600	−449.9603	600.0080	300.0284
1388.8600	56.5600	−532.4400	−449.9826	300.1623	300.0306
1381.8900	−243.3700	−534.9800	−449.9776	0.2605	300.0453
1376.7600	−466.1520	−536.9800	−449.9721	−222.6143	300.0191
1374.9600	−542.8600	−537.7000	−449.9723	−299.3827	300.0141
${\tilde{X}}^{UR}$			${\hat{X}}^{UR}$		
***x*, mm**	***y*, mm**	***z*, mm**	***x*, mm**	***y*, mm**	***z*, mm**
−299.1941	600.6059	748.8994	−299.4489	600.5320	749.7033
−299.5745	300.5496	749.0396	−299.8734	300.2792	749.6301
−299.5501	1.1412	749.3286	−299.9692	0.0865	749.4284
−301.7034	−298.4790	749.5021	−300.1888	−299.7125	749.3410
−301.2012	−298.9596	449.4663	−300.1222	−299.8256	449.4001
−299.9751	0.5753	449.0687	−300.0666	−0.0251	449.3135
−300.2272	299.8920	449.0341	−299.9672	300.2473	449.6485
−299.6391	600.0386	448.9144	−299.4983	600.5386	449.6498
−449.1337	600.5285	298.7817	−449.3979	600.7467	299.3194
−449.7079	300.5363	298.9477	−449.8548	300.5789	299.3242
−450.0987	0.8905	299.1044	−450.0003	0.48914	299.3347
−450.6372	−221.6012	299.28724	−450.1226	−222.2956	299.3356
−450.8788	−298.2697	299.33644	−450.2056	−299.0152	299.3373

## Data Availability

There is no data set associated with the paper.
